# Ruptured Sinus of Valsalva Aneurysm into the Left Atrium with Multiple Fistulous Communications: A Rare Cause of Heart Failure

**DOI:** 10.1155/2015/627946

**Published:** 2015-12-27

**Authors:** Yashwant Agrawal, Rakshita Chandrashekhar, Jerry W. Pratt, Maria D. Cole, Sreenivas Kamath, Jagadeesh K. Kalavakunta

**Affiliations:** ^1^Department of Internal Medicine/Pediatrics and Department of Internal Medicine, Western Michigan University Homer Stryker School of Medicine, Kalamazoo, MI 49048, USA; ^2^Department of Cardiology and Cardiothoracic Surgery, Borgess Medical Center, Kalamazoo, MI 49048, USA

## Abstract

Ruptured noncoronary sinus of valsalva aneurysm with fistulous connections to multiple cardiac chambers has not been reported previously. We report a 66-year-old man who presented with worsening cough and exertional dyspnea. Transesophageal echocardiogram confirmed a large aneurysm involving the noncoronary cusp of the aortic sinus with aneurysmal extension to the left atrium. There were also two fistulous communications with the left atrium and one small fistulous connection with the right atrium. Open-heart surgery with aortic root replacement and reimplantation of coronary arteries along with primary closure and repair of aorta to the left atrial fistula was performed.

## 1. Introduction

Though ruptured sinus of valsalva aneurysm is a rare entity when it occurs, emergent surgical intervention should be the treatment of choice as patients can deteriorate rapidly and if required prophylactic repair of other structures such as impending aneurysm should be performed, as in our case, to prevent future catastrophes.

## 2. Case History

**Figure 1:**
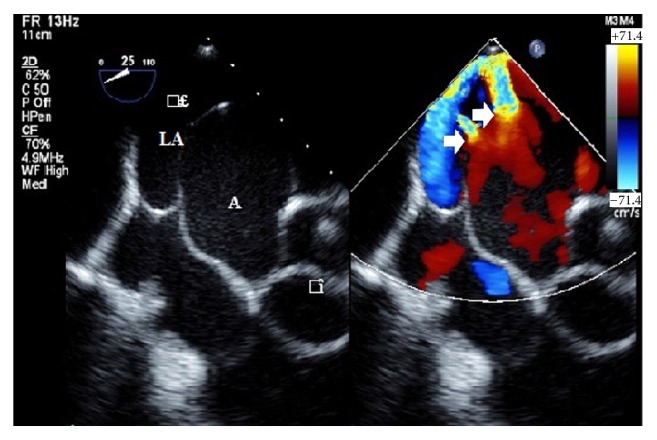
TEE showing fistulous communication (arrow) between sinus of valsalva aneurysm (A) and left atrium (LA).

A 66-year-old man presented to the emergency room with worsening cough, exertional dyspnea, and orthopnea for 3 weeks. Physical examination was remarkable for a grade III/VI continuous murmur at the second right upper sternal border. Initial lab values were remarkable for troponin 0.1 ng/mL (normal, 0–0.16 ng/mL) and brain natriuretic peptide 761 ng/mL (normal, 0–450 ng/L). He was placed on intravenous furosemide, which improved his symptoms. A transthoracic echocardiogram showed marked dilatation of sinuses of valsalva and an aneurysm, which seemed to have ruptured into the left atrium (LA). For better assessment, a transesophageal echocardiogram (TEE) was performed which showed large aneurysm involving the noncoronary cusp of the aortic sinus of valsalva extending into LA (Figure 1). The gradient across the communication was 65 mmHg. Two fistulous connections with LA were also appreciated along with a small fistulous connection with right atrium. The aortic valve was eufunctional without any stenosis or regurgitation on TEE. Two days later, he developed acute dyspnea and went into cardiogenic shock. His vitals were significant for blood pressure of 80/46 mmHg, heart rate 128 bpm, and respiratory rate 30/minute with saturation of 78%. His lab values were remarkable for creatinine increasing to 2.8 mg/dL from 1.3 mg/dL (normal, 0.6–1.2 mg/dL) and brain natriuretic peptide 1500 ng/mL from 764 ng/mL. He underwent emergent sinus of valsalva aneurysm (SVA) repair and aortic root replacement with 25 mm freestyle aortic root. During the procedure, a large SVA of the noncoronary sinus was noted, herniating into the LA. At the end of the windsock, there were two holes about 5–8 mm in diameter contributing significantly to the aortoleft atrial shunting. The left and the right coronary sinus had significant degeneration of the walls comprising only a single layer of cells. There were fenestrations in the left and noncoronary cusps of the aortic valve in close proximity with the aortic valve leaflets. With high risk of developing aneurysms from these sinuses and fenestrations in 2 of the 3 aortic cusps in close proximity with the aortic leaflets, replacement of the whole root with freestyle root 25 mm was performed with aortic valve replacement. The SVA sac was suture-ligated and base was closed by sewing the aortic annulus at the noncoronary sinus side to the aortic sinus wall. The aortic sinus wall and aneurysmal wall also had major degeneration comprising of a one-cell layer thickness with underlying muscle layer clearly seen. Patient recovered well and was sent to cardiac rehabilitation.

## 3. Discussion

SVA is a rare cardiac malformation that can be congenital or acquired. 0.09% of SVAs were reported in cadavers of a large autopsy series and ranged between 0.14 and 0.23% [1].

Congenital SVAs are typically silent clinically and detected in routine echocardiograms. However, symptomatic presentations related to the compression of adjacent cardiac structures or intracardiac shunting caused by ruptured of the SVA into the right side of the heart also occur, especially in older patients. Most commonly, SVAs originate from the right sinus of valsalva, accounting for about 75% of the cases, while noncoronary (10–30%) and left sinuses (<5%) are exceedingly accounting for the rest.

The most common complication is rupture into the atrium or ventricle, and, very rarely, towards the left chambers, causing left-to-right shunting or aortic valve insufficiency with congestive heart failure and the need for urgent surgical resolution. In two of the largest single center studies, no fistulous communication with the LA was identified from the noncoronary sinus [2, 3]. The few cases of reported ruptured noncoronary SVA into the LA have been at least 40 years back [4].

Open-heart surgery with or without aortic valve replacement is the optimal intervention with transcatheter device closure of a ruptured SVA also gaining popularity [5].

Surgical interventions for SVA repair include approach through the chamber involved via an aortotomy or a collective approach. Performing an aortotomy needs to be determined on a case-by-case basis with routine aortotomy being debatable [6–10].

The type of surgery for a SVA is multifactorial dependent: the site of aneurysmal rupture, aortic valve involvement, aneurysmal orifice definition, and coexistence of other cardiac anomalies. For a ruptured SVA, the recommended surgical approach is patch closure of the aneurysmal orifice with aortic valve sparing [5]. Direct closure would be a choice for smaller aneurysms [11, 12]. Functional status of the aortic valve mainly determines the need for aortic valve replacement or valve-sparing procedures like remodeling or reimplantation [13]. Reports requiring prosthetic replacement of the entire aortic root, reimplantation of the coronary arteries, or both have been described. Surgical success is mainly determined by preexistent endocarditis and sepsis with higher mortality rates (3.9%) in such cases compared to noninfected cases (1%). 10-year survival rate of a successfully repaired ruptured SVA is about 90–95%, irrespective of the procedure performed [14].

The sinus of valsalva aneurysm in our patient was most likely acquired from atherosclerosis given the extensive nature of degenerative changes seen in the adjacent cardiac structures. Since he did not have a prior echocardiogram, there is no sure way of knowing if the aneurysm was indeed acquired or was congenital with a late presentation. In our case, an open-heart surgery with transverse aortotomy was performed which included a #25 freestyle inclusion root replacement, reimplantation of coronary arteries, and primary closure repair of aorta to the left atrial fistula given the extensive nature of degenerated vessels with fenestrations in 2 of the 3 aortic cusps.
